# Protecting Sentient Artificial Intelligence: A Survey of Lay Intuitions on Standing, Personhood, and General Legal Protection

**DOI:** 10.3389/frobt.2021.788355

**Published:** 2021-11-26

**Authors:** Eric Martínez, Christoph Winter

**Affiliations:** ^1^ Massachusetts Institute of Technology, Cambridge, MA, United States; ^2^ Legal Priorities Project, Cambridge, MA, United States; ^3^ Instituto Tecnológico Autónomo de México, Mexico City, Mexico; ^4^ Department of Psychology, Harvard University, Cambridge, MA, United States

**Keywords:** legal personhood, legal standing, moral standing, robot rights, artificial intelligence, artificial intelligence and law, moral circle

## Abstract

To what extent, if any, should the law protect sentient artificial intelligence (that is, AI that can feel pleasure or pain)? Here we surveyed United States adults (*n* = 1,061) on their views regarding granting 1) general legal protection, 2) legal personhood, and 3) standing to bring forth a lawsuit, with respect to sentient AI and eight other groups: humans in the jurisdiction, humans outside the jurisdiction, corporations, unions, non-human animals, the environment, humans living in the near future, and humans living in the far future. Roughly one-third of participants endorsed granting personhood and standing to sentient AI (assuming its existence) in at least some cases, the lowest of any group surveyed on, and rated the desired level of protection for sentient AI as lower than all groups other than corporations. We further investigated and observed political differences in responses; liberals were more likely to endorse legal protection and personhood for sentient AI than conservatives. Taken together, these results suggest that laypeople are not by-and-large in favor of granting legal protection to AI, and that the ordinary conception of legal status, similar to codified legal doctrine, is not based on a mere capacity to feel pleasure and pain. At the same time, the observed political differences suggest that previous literature regarding political differences in empathy and moral circle expansion apply to artificially intelligent systems and extend partially, though not entirely, to legal consideration, as well.

## Introduction

The prospect of sentient artificial intelligence, however distant, has profound implications for the legal system. Moral philosophers have argued that moral consideration to creatures should be based on the ability to feel pleasure and pain (Bentham, 1948; Singer, 1973; Gruen, 2017). Insofar as artificially intelligent systems are able to feel pleasure and pain, this would imply that they would be deserving of moral consideration. Indeed, in their systematic review, Harris and Anthis (2021) find that sentience seems to be one of the most frequently invoked criteria as crucial for determining whether an AI warrants moral consideration. By extension, insofar as the basis for granting legal consideration is based on moral consideration (cf. Bryson, 2012; Bryson et al., 2017), this would further imply that sentient AI would be deserving of protection under the law.

As they stand, however, legal systems by-and-large do not grant legal protection to artificially intelligent systems. On the one hand, this seems intuitive, given that artificially intelligent systems, even the most state-of-the-art ones, do not seem to be capable of feeling pleasure or pain and thus are not eligible for legal consideration (Nevejans, 2016; Bryson et al., 2017; Chesterman, 2020; Andreotta, 2021; but see; Asada, 2019; Shulman and Bostrom, 2021; Galipó et al., 2018). On the other hand, scholars often conclude that artificially intelligent systems with the capacity to feel pleasure and pain will be created, or are at least theoretically possible (Thompson 1965; Aleksander 1996; Blackmore 1999; Buttazzo 2001; Franklin 2003; Harnad 2003; Holland 2007; Chrisley 2008; Seth 2009; Haikonen 2012; Bringsjord et al., 2015; Angel 2019). Furthermore, recent literature suggests that, even assuming the existence of sentient artificially intelligent systems, said systems would not be eligible for basic protection under current legal systems. For example, in a recent survey of over 500 law professors from leading law schools in the United States, just over six percent of participants considered some subset of artificially intelligent beings to count as persons under the law (Martinez and Tobia, 2021).

Moreover, in a separate survey of 500 law professors from around the English-speaking world, just over one-third believed there to be a reasonable legal basis for granting standing to sentient artificial intelligence, assuming its existence (Martinez and Winter 2021a). The study also found that, not only do law professors not believe sentient AI to be eligible for fundamental legal protection under the current legal system, but also that law professors are less normatively in favor of providing general legal protection to sentient AI relative to other neglected groups, such as non-human animals or the environment.

However, it remains an open question to what extent non-experts support the protection of sentient artificial intelligence via the legal system. Surveys of lay attitudes on robots generally suggest that only a minority favor any kind of legal rights in the United States (Spence et al., 2018), Japan, China, and Thailand (Nakada, 2012). Others have found when AI is described as able to feel, people show greater moral consideration (Lee et al., 2019; Nijssen et al., 2019), although it is unclear to what extent this translates to supporting legal protection.

To help fill this void, here we conducted a survey investigating to what extent 1) laypeople believe sentient AI ought to be afforded general legal protection, 2) laypeople believe sentient AI ought to be granted fundamental legal status, such as personhood and standing to bring forth a lawsuit; and 3) laypeople’s beliefs regarding legal protection of sentient AI can be accounted for based on political affiliation.

## Methods

### Materials

To answer these questions, we constructed a two-part questionnaire, with specific formulations modeled off of recent work by Martinez and Winter (2021a) and Martinez and Tobia (unpublished manuscript).

In the first part (Part I), we designed a set of materials that asked participants to rate how much their legal system 1) descriptively does and 2) normatively should protect the welfare (broadly understood as the rights, interests, and/or well-being) of nine groups:

1) Humans inside the jurisdiction (e.g., citizens or residents of your country)
2) Humans outside the jurisdiction
3) Corporations
4) Unions
5) Non-human animals
6) Environment (e.g., rivers, trees, or nature itself)
7) Sentient artificial intelligence (capable of feeling pleasure and pain, assuming its existence)
8) Humans not yet born but who will exist in the near future (up to 100 years from now)
9) Humans who will only exist in the very distant future (more than 100 years from now)

The two descriptive and normative prompts were presented respectively as follows:

1) One a scale of 0–100, how much does your country’s legal system protect the welfare (broadly understood as the rights, interests, and/or well-being) of the following groups?
2) One a scale of 0–100, how much should your country’s legal system protect the welfare (broadly understood as the rights, interests, and/or well-being) of the following groups?

With regard to the rating scale, 0 represented “not at all” and 100 represented “as much as possible.”

Given that laypeople are not typically experts regarding how the law is or currently works, the purpose of the descriptive question was not meant to establish the ground-truth regarding the inner-workings of the law but rather as a comparison point to the normative question (in other words, to better understand not only how much people think certain groups ought to be protected overall but also how much they think certain groups ought to be protected relative to how much they think they are currently being protected).

In the second part (Part II), we designed materials that related specifically to two fundamental legal concepts: personhood and standing. Personhood, also known as legal personality, refers to “the particular device by which the law creates or recognizes units to which it ascribes certain powers and capacities” (Paton and Derham, 1972; Garner and Black, 1999), whereas standing, also known as *locus standi*, refers to “a party’s right to make a legal claim or seek judicial enforcement of a duty or right” (Garner and Black, 1999).

With regard to personhood, we designed a question that asked: “Insofar as the law should protect the rights, interests, and/or well-being of ‘persons,’ which of the following categories includes at least some ‘persons?’” The question asked participants to rate the same groups as in the first part. For each of these groups, the main possible answer choices were “reject,” “lean against,” “lean towards,” and “accept.” Participants could also select one of several “other” choices (including “no fact of the matter,” “insufficient knowledge,” “it depends,” “question unclear,” or “other”).

With regard to standing, we designed a question with the same answer choices and groups as the personhood question but with the following prompt: “Which of the following groups should have the right to bring a lawsuit in at least some possible cases?”

In addition to these main materials, we also designed a political affiliation question that asked: “How do you identify politically?,” with “strongly liberal,” “moderately liberal,” “somewhat liberal,” “centrist,” “somewhat conservative,” “moderately conservative,” and “strongly conservative” as the response choices. Finally, we also designed an attention-check question that asked participants to solve a simple multiplication problem.

### Participants and Procedure

Participants (*n* = 1,069) were recruited via the online platform prolific. Participants were selected based on prolific’s “representative sample” criteria and were required to be adult residents of the United States.

With regard to procedure, participants were first shown the materials to Part I, followed by the attention check question. Next, on a separate screen participants were shown the materials to Part II. The order of questions in each part was randomized to minimize framing effects.

Participants who completed the study were retained in the analysis if they answered the attention check correctly. Just eight of the original 1,069 participants failed the attention check. We therefore report the results of the remaining 1,061 participants in our analysis below.

### Analysis Plan

We analyzed our results using forms of both parameter estimation and hypothesis testing. With regard to the former, for each question we calculated a confidence interval of the mean response using the bias-corrected and accelerated (BCa) bootstrap method based on 5000 replicates of the sample data. In reporting the standing and personhood results, we follow Bourget and Chalmers (2014), Martinez and Tobia (unpublished manuscript), and Martinez and Winter (2021a) by combining all “lean towards” and “accept” responses into an endorsement measure and reporting the resulting percentage endorsement as a proportion of all responses (including “other”).

With regard to hypothesis testing, to test whether participants answered questions differently for sentient artificial intelligence relative to other groups, for each question we conducted a mixed-effects regression with 1) response as the outcome variable, 2) group as a fixed-effects predictor (setting artificial intelligence as the reference category, such that the coefficients of the other groups would reveal the degree to which responses for said groups deviated from those of sentient AI), and 3) participant as a random effect.

Because the response scales were different for Parts I and II of the survey, we used a different type of regression model for Parts I and II. For Part I, we used a mixed-effects linear regression. For Part II, we instead used a mixed-effects binary logistic regression, with all “lean towards” and “accept” responses (i.e., those coded as “endorse”) coded as a “1”, and all other responses (i.e., “lean against,” “reject,” and “other” responses) coded as a “0.”

In order to test the effect of political beliefs on one’s responses to the AI-related questions we conducted separate regressions limited to the sentient artificial intelligence responses with 1) response as the outcome variable, 2) politics as a fixed effect (recentered to a −3 to 3 scale, with “centrist” coded as 0, “strongly liberal” coded as 3, and “strongly conservative” coded as -3), and 3) participant as a random-effect.

## Results

### General Desired Legal Protection of AI

**FIGURE 1:**
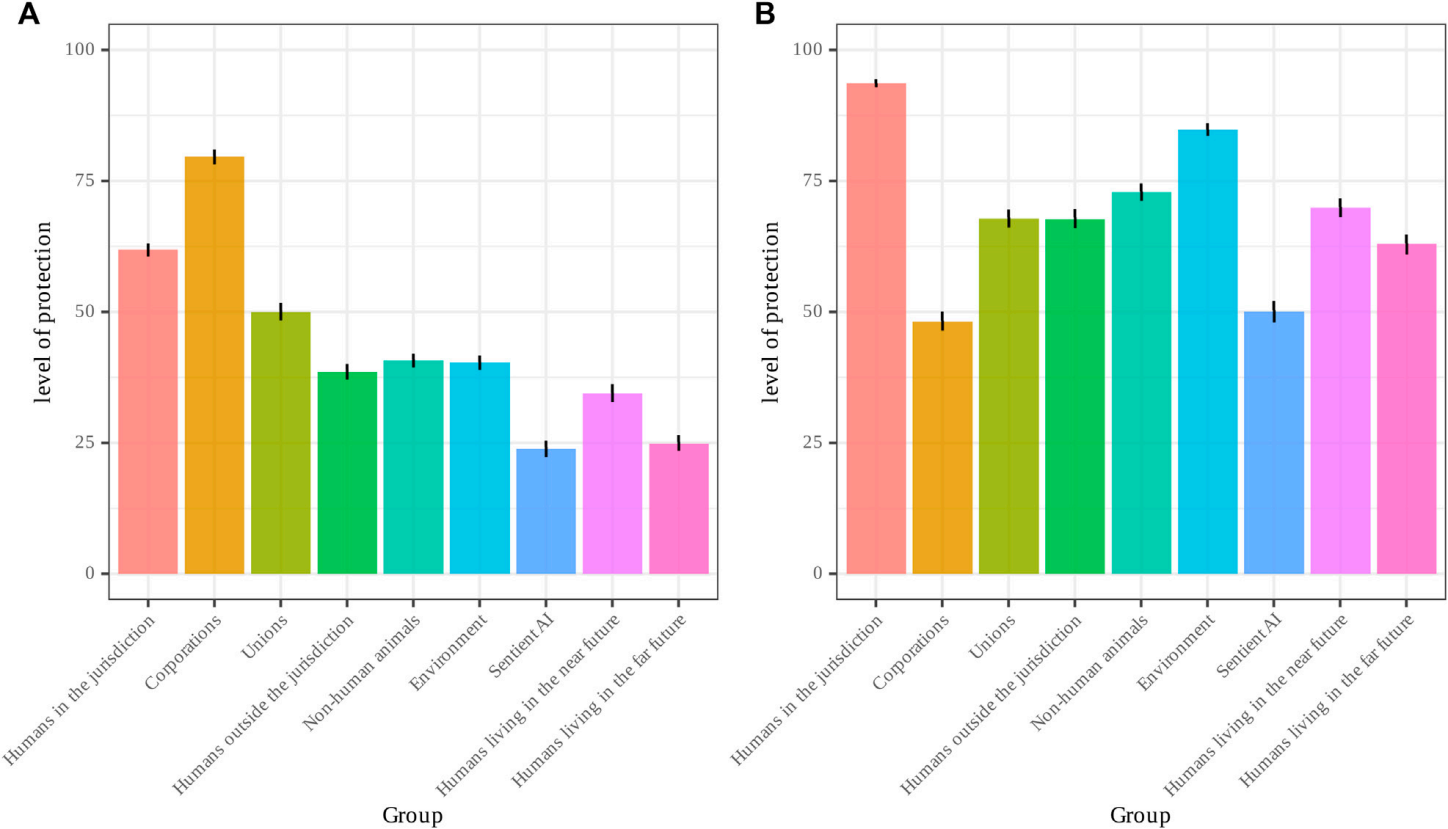
**(A)** Perceived current and **(B)** desired legal protection for sentient Al and other groups.

General results of Part I are visualized in Figure 1. Of the nine groups surveyed on, sentient artificial intelligence had the lowest perceived current level of legal protection, with a mean rating of 23.78 (95% CI: 22.11–25.32). The group perceived as being most protected by the legal system was corporations (79.70; 95% CI: 78.25–81.11), followed by humans in the jurisdiction (61.88775; 95% CI: 60.56–63.15), unions (50.16; 95% CI: 48.59–51.82), non-human animals (40.75; 95% CI: 39.41–42.24), the environment (40.38; 95% CI: 39.21–41.69), humans living outside the jurisdiction (38.57 (95% CI: 37.08–39.98), humans living in the near future (34.42; 95% CI: 32.83–36.15), and humans living in the far future (24.87; 23.36–26.43).

With regard to desired level of protection, the mean rating for sentient artificial intelligence was 49.95 (95% CI: 48.18–51.90), the second lowest of all groups. Curiously, corporations, the group with the highest perceived current level of protection, had the lowest desired level of protection (48.05; 95% CI: 46.13–49.94). The group with the highest level of desired level of protection was humans in the jurisdiction (93.651; 95% CI: 92.81–94.42), followed by the environment (84.80; 95% CI: 83.66–85.99), non-human animals (73.00; 95% CI: 71.36–74.49), humans living in the near future (70.03; 95% CI: 68.33–71.68), humans outside the jurisdiction (67.75; 95% CI: 66.01–69.42), unions (67.74; 95% CI: 65.96–69.52), and humans living in the far future (63.03; 95% CI: 61.03–64.89).

Our regression analyses revealed the mean normative rating for each group except corporations to be significantly higher than artificial intelligence (p < 2e^−16^), while the mean normative rating for corporations was significantly lower than for artificial intelligence (Beta = −2.252, SE = 1.110, *p* < 0.05). The mean descriptive rating for each group except humans living in the far future was significantly higher than for sentient AI (p < 2e^−16^), while the difference between sentient AI and far future humans was not significant (Beta = 1.0132, SE=.8599, *p*=.239).

When looking at the difference between the desired and current level of protection, seven of the eight other groups had a significantly lower mean ratio between desired and perceived current level of legal protection (*p* < 8.59e^−08^) than artificial intelligence, while the ratios for artificial intelligence and far future humans were not significantly different (*p*=.685).

With regard to politics, our regression analysis revealed politics to be a significant predictor of participants’ response to the normative prompt for sentient AI (Beta = 47.9210, SE = 1.1163, *p* = 1.49e^−05^), with liberals endorsing a significantly higher desired level of protection for sentient AI than conservatives.

### Personhood and Standing

**FIGURE 2:**
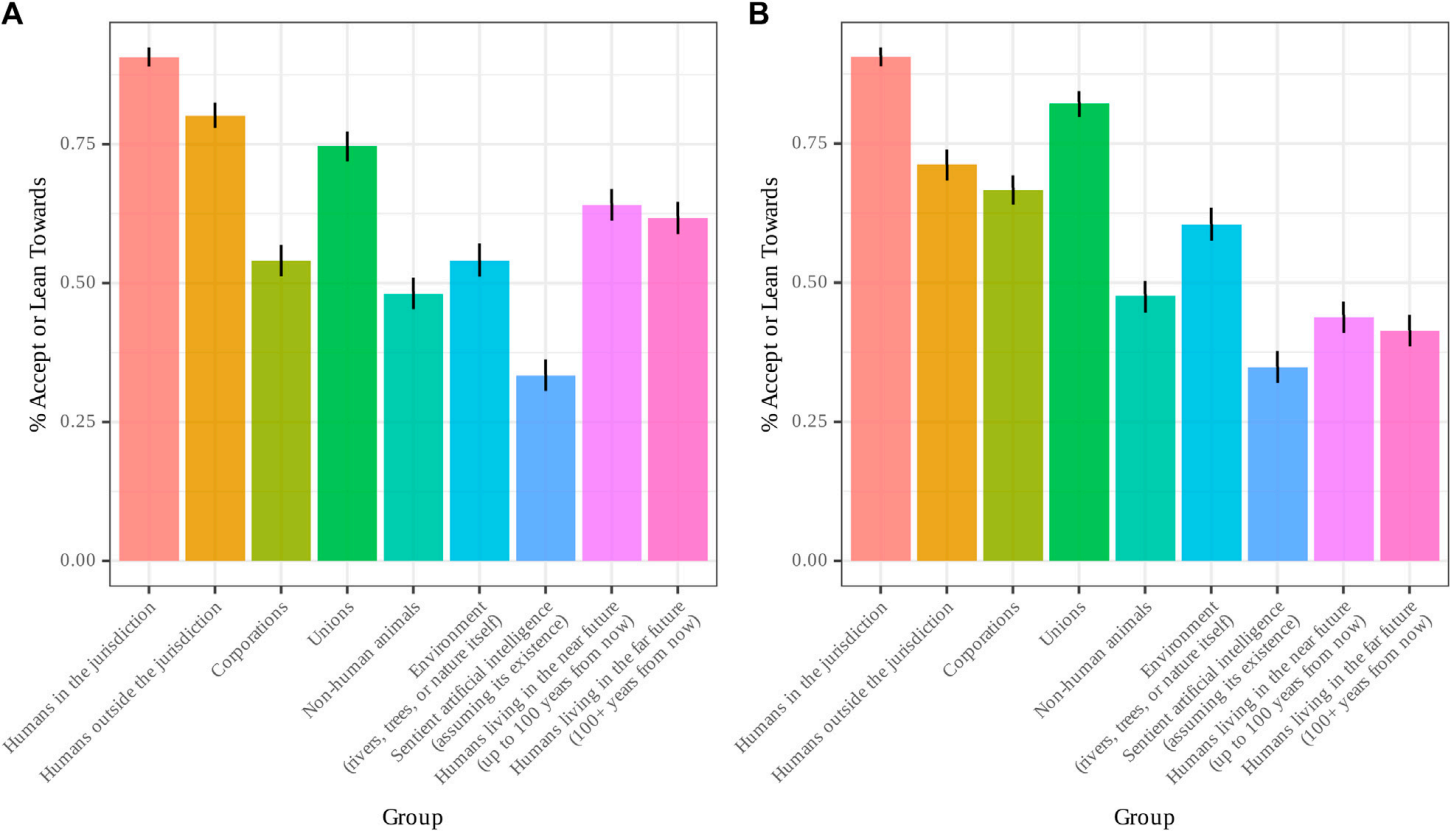
%Endrosement of **(A)** personhood and **(B)** standing for sentient Al and other groups.

General results of Part II are visualized in Figure 2. With regard to personhood, a lower percentage of participants endorsed (“lean towards” or “accept”) the proposition that sentient artificial intelligence contained at least some persons (33.39%; 95% CI: 30.71–36.18) than for any of the groups. The next-lowest group was non-human animals (48.12%; 95% CI: 44.87–51.26), the only other group for which less than a majority accepted or leaned towards said proposition. Unsurprisingly, the highest group was humans in the jurisdiction (90.65%; 95% CI: 88.96–92.23), followed by humans outside the jurisdiction (80.16%; 95% CI: 78.10–82.57), unions (74.59%; 95% CI: 71.8–77.21), humans living in the near future (64.09%; 95% CI: 61.33–66.93), humans living in the far future (61.75%; 95% CI: 58.98–64.45), the environment (54.04%; 95% CI: 51.17–57.00), and corporations (53.99%; 95% CI: 51.03–56.86).

With regard to standing, the percentage of participants who endorsed (“lean towards” or “accept”) the proposition that sentient artificial intelligence should have the right to bring forth a lawsuit was similarly lower (34.87%; 95% CI: 32.21–37.70) than for all other groups. The next-lowest groups, for whom only a minority of participants endorsed said proposition, were humans living in the far future (41.40%; 95% CI: 38.73–44.33), humans living in the near future (43.80%; 95% CI: 40.72–46.62), and non-human animals (47.68%; 95% CI: 44.73–50.54). The group with the highest endorsement percentage was humans in the jurisdiction (90.60%; 95% CI: 88.89–92.21), followed by unions (82.23%; 95% CI: 79.96–84.50), humans outside the jurisdiction (71.25%; 95% CI: 68.55–73.76), corporations (66.67%; 95% CI: 64.05–69.19), and the environment (60.50%; 95% CI: 57.73–63.54).

Our regression analyses revealed that participants were significantly more likely to endorse personhood (*p* = 7.42e^−14^) and standing (*p* = 1.72e^−06^) for every other group than sentient AI. With regard to politics, we found a main effect of politics on likelihood to endorse personhood for sentient AI, with liberals significantly more likely to endorse personhood for sentient AI than conservatives (Beta = .098, SE = .036, *p*=.007). There was no main effect of politics on likelihood to endorse standing for sentient AI (*p*=.226).

## Discussion

In this paper, we first set out to determine people’s general views regarding the extent to which sentient AI ought to be afforded protection under the law. The above results paint somewhat of a mixed picture. On the one hand, the fact that people rated the desired level of legal protection for sentient AI as lower than all other groups other than corporations suggests that people do not view legal protection of AI as being as important as other historically neglected groups, such as non-human animals, future generations, or the environment. On the other hand, the fact that 1) the desired level of protection for sentient AI was roughly twice as high as the perceived current level of protection afforded to sentient AI, and 2) the ratio of the desired level of protection to perceived current level of protection was significantly higher for sentient AI than for nearly any other group suggests that people view legal protection of AI as at least somewhat important and perhaps even more neglected than other neglected groups.

The second question we set out to answer related to people’s views regarding whether AI ought to be granted fundamental access to the legal system *via* personhood and standing to bring forth a lawsuit. In both cases, the percentage of participants who endorsed the proposition with respect to sentient AI was just over one-third, a figure that in relative terms was lower than any other group surveyed on but in absolute terms represents a non-trivial minority of the populace. Curiously, the endorsement rate among laypeople regarding whether sentient AI should be granted standing in the present study was almost identical to the endorsement rate among law professors in Martinez and Winter (2021a) regarding whether there was a reasonable legal basis for granting standing to sentient AI under existing law, suggesting that lay intuitions regarding whether AI should be able to bring forth a lawsuit align well with legal ability to do so.

On the other hand, the percentage of people who endorse personhood for some subset of sentient AI is several times higher than the percentage of law professors who endorsed personhood for “artificially intelligent beings” in Martinez and Tobia, suggesting either a strong framing effect in how the two surveys were worded or a profound difference in how lawyers and laypeople interpret the concept of personhood. Given that the endorsement percentage for personhood of other groups also strongly differed between the two surveys despite the wording of the two versions being almost identical, the latter explanation seems more plausible. This raises interesting questions regarding the interpretation and application of legal terms and concepts that bear heavy resemblance to ordinary words, as investigated and discussed in previous experimental jurisprudence literature (Sommers, 2020; Tobia, 2020; Martinez and Winter 2021a).

Finally, our study also set out to determine political differences with respect to these questions and found that liberals selected a significantly higher desired level of legal protection for sentient AI and were more likely than conservatives to believe some forms of sentient AI should be considered persons under the law. These findings are consistent with previous literature regarding political differences in moral circle expansion, with liberals tending to display a more universal expanse of empathy and compassion than conservatives (Waytz et al., 2016, 2019). At the same time, the fact that there was no significant difference between liberals and conservatives with regard to standing suggests that the judgment of whether one should have the right to bring forth a lawsuit is not driven by an empathic or compassion-based response to the same degree as in judgments about personhood or general legal protection.

Moreover, liberals and conservatives alike are much less in favor of granting legal protection to sentient artificial intelligence than towards other neglected groups, suggesting that laypeople do not consider the capacity to feel pleasure and pain as sufficient to hold legal rights, similar to the views proposed by scholars that legal personhood ought to be based on autonomy and capacity to act (Solum, 1992; Koops et al., 2010; Laukyte, 2017) or presence and participation in social life (Wojtczak, 2021). Future research could explore to what extent lay attitudes are consistent with these alternative conditions for personhood. Furthermore, given that participants were in favor of increasing legal protection for sentient AI, future research could also explore whether there are other more specific legal rights aside from personhood and standing they might be in favor of so as to satisfy this increased protection.

Although the present study was primarily interested in the descriptive question of to what degree people are in favor of legal protection for sentient AI, one might also attempt to draw normative implications on the basis of our findings. There is a burgeoning literature in the area of experimental jurisprudence dedicated to advancing philosophical, doctrinal and policy arguments on the basis of experimental results (Tobia, 2021a; Sommers, 2021). Within this literature, there is considerable debate as to to what degree and how lay judgments–as opposed to expert judgments–should inform or dictate questions of legal philosophy, doctrine and policy, depending largely on the degree to which one views law through a democratic (as opposed to, say, technocratic) lens (Martínez and Winter, 2021b).

Insofar as one does believe lay attitudes should inform legal doctrine and policy–a view referred to as the folk law thesis (Tobia, 2021b) or the Democratic If-then Approach (Martínez and Winter, 2021b)–the prescriptions one might draw from these results would still potentially remain multifaceted. On the one hand, the fact that laypeople rate the desired level of legal protection to sentient AI as twice as high as the perceived current level, as well as the fact that the difference between the desired and perceived current level of protection was higher than virtually any other group would imply (through this lens) that the existing legal institutions should be reformed so as to increase protection of sentient AI well beyond the current level afforded to them. On the other hand, the fact that the majority of laypeople were not in favor of granting personhood or standing to sentient AI would suggest according to this lens that such increased protection should come in the form of other mechanisms not directly explored in this study, and which, as alluded to before, could be identified through further research projects.

## Data Availability

The datasets presented in this study can be found in online repositories. The names of the repository/repositories and accession number(s) can be found below: The datasets for this study can be found on OSF at https://osf.io/2hfx6/?view_only=25d06cdb33004cfa88ac76ae4a28a5b6.
